# Contrasting Genomic Evolution Between Domesticated and Wild *Kluyveromyces lactis* Yeast Populations

**DOI:** 10.1093/gbe/evad004

**Published:** 2023-01-13

**Authors:** Anne Friedrich, Jean-Sébastien Gounot, Andreas Tsouris, Claudine Bleykasten, Kelle Freel, Claudia Caradec, Joseph Schacherer

**Affiliations:** Université de Strasbourg, CNRS, GMGM UMR 7156, Strasbourg 67000, France; Université de Strasbourg, CNRS, GMGM UMR 7156, Strasbourg 67000, France; Université de Strasbourg, CNRS, GMGM UMR 7156, Strasbourg 67000, France; Université de Strasbourg, CNRS, GMGM UMR 7156, Strasbourg 67000, France; Université de Strasbourg, CNRS, GMGM UMR 7156, Strasbourg 67000, France; Université de Strasbourg, CNRS, GMGM UMR 7156, Strasbourg 67000, France; Université de Strasbourg, CNRS, GMGM UMR 7156, Strasbourg 67000, France; Institut Universitaire de France (IUF), Paris 75005, France

**Keywords:** genome evolution, population genomics, domestication, introgression, yeast, *Kluyveromyces lactis*

## Abstract

The process of domestication has variable consequences on genome evolution leading to different phenotypic signatures. Access to the complete genome sequences of a large number of individuals makes it possible to explore the different facets of this domestication process. Here, we sought to explore the genome evolution of *Kluyveromyces lactis*, a yeast species well known for its involvement in dairy processes and also present in natural environments. Using a combination of short- and long-read sequencing strategies, we investigated the genomic variability of 41 *K. lactis* isolates and found that the overall genetic diversity of this species is very high (θ_w_ = 3.3 × 10^−2^) compared with other species such as *Saccharomyces cerevisiae* (θ_w_ = 1.6 × 10^−2^). However, the domesticated dairy population shows a reduced level of diversity (θ_w_ = 1 × 10^−3^), probably due to a domestication bottleneck. In addition, this entire population is characterized by the introgression of the *LAC4* and *LAC12* genes, responsible for lactose fermentation and coming from the closely related species, *Kluyveromyces marxianus*, as previously described. Our results highlighted that the *LAC4*/*LAC12* gene cluster was acquired through multiple and independent introgression events. Finally, we also identified several genes that could play a role in adaptation to dairy environments through copy number variation. These genes are involved in sugar consumption, flocculation, and drug resistance, and may play a role in dairy processes. Overall, our study illustrates contrasting genomic evolution and sheds new light on the impact of domestication processes on it.

**Significance**

Domestication is a human-related process that has shaped the genome of many species and can affect genome evolution in many ways. Through a population genomic survey, we sought to explore this aspect in the yeast *Kluyveromyces lactis*, a species well known for its involvement in dairy processes and also present in natural environments. We found that the domesticated dairy population is characterized by a reduced level of diversity and is punctuated by multiple introgression events of the *LAC* gene cluster, responsible for lactose fermentation, from the sister species *Kluyveromyces marxianus*. Our study sheds new light on the impact of domestication processes by illustrating differential genomic evolution between populations.

## Introduction

Domestication is a human-related process that shaped the genome of many species. Indeed, by selecting organisms for desirable traits for millennia, humans have acted unconsciously on the evolution of these genomes. Domesticated species, therefore, represent valuable models for the study of adaptive divergence. While the domestication of plants and animals has always been carried out on purpose, the selection for microorganisms was first conducted unintentionally, before being better controlled. Indeed, there is evidence for fermented foods and beverages since the Neolithic, based first on naturally existing microbiota, that predates the use of backslopping technics, far before the discovery of microbes in the late 17th century. In this context, multicellular fungi and yeasts have played a predominant role (Dupont et al., 2017), and access to whole-genome sequencing data of a large number of individuals having undergone these processes allowed to gain insight into the genomic adaptation footprints at the species level.

Particular interest has been taken in fungal species such as *Aspergillus oryzae*, used for rice and soy fermentation (Gibbons et al., 2012), as well as *Penicillium* species such as *P. roqueforti* and *P. camemberti*, both used for the maturation of cheese (Cheeseman et al., 2014; Ropars et al., 2020). Genomic exploration of these two species revealed that their adaptation to the cheese environment was associated with recent horizontal transfers of large genomic regions carrying crucial metabolic genes. However, distinct domestication events can coexist in the same species, generally leading to divergent phenotypic traits (Ropars et al., 2015, 2020). Much of the attention has also been focused on the yeast *Saccharomyces cerevisiae*, a key driver of several industrial fermentation processes. Indeed, the exploration of thousands of complete genomes over the past decade provides evidence for various independent and lineage-specific domestication events within this species, leading to different evolutionary trajectories (Duan et al., 2018; Gallone et al., 2016; Peter et al., 2018; Strope et al., 2015). The beer and bakery populations have been shown to be polyphyletic and diverse at the nucleotide and ploidy levels, for example (Bigey et al., 2021; Gallone et al., 2016; Peter et al., 2018; Saada et al., 2022). In contrast, the wine and sake populations are monophyletic and much less genetically diverse. Genomic processes detected as conferring the desired properties of domesticated strains are diverse and include copy number variants (CNVs), horizontal gene transfer, large structural variants, as well as single nucleotide polymorphisms (SNPs) (Dunn et al., 2012; Gonçalves et al., 2016; Lee et al., 2022; Legras et al., 2018; Peltier et al., 2019).

In fact, *Saccharomyces cerevisiae* is no exception and many yeast species have undergone domestication processes (Almeida et al., 2014; Banjara et al., 2015; Hranilovic et al., 2018; Varela et al., 2019). In this context, *K. lactis* represents an interesting species. Considered as an alternative yeast model in genetics and physiology (Rodicio & Heinisch, 2013; Schaffrath & Breunig, 2000), attractive for biotechnological processes such as the production of pharmaceuticals (Spohner et al., 2016) and heterologous proteins (Van Ooyen et al., 2006), this yeast is especially well known for its ability to ferment lactose. *Kluyveromyces lactis* represents, together with its sister species, *K. marxianus*, one of the leading yeast contributors to dairy products. In *K. lactis*, the lactose assimilation process relies on a well-known pathway comprising the *LAC4* and *LAC12* genes, which encode a ß-galactosidase and a lactose permease, respectively, as well as the galactose-lactose regulatory genes (*LAC9* and *GAL80*) and the galactose genes (*GAL1, GAL7,* and *GAL10*) (Dickson & Riley, 1989; Naumov et al., 2006). However, this yeast can also be isolated from natural environments, mainly from insects and tree exudates and these natural isolates are not able to use lactose as a carbon source. Based on these physiological and ecological characteristics, *K. lactis* was divided into two varieties: *K. lactis* var. *lactis* and *K. lactis* var. *drosophilarum* (Naumov et al., 2014; Sidenberg & Lachance, 1986). The distinction between the two varieties was supported by sequence evidence, as shown by the analysis of the 5.8S-ITS rDNA of multiple samples, but the variety *K. lactis* var. *drosophilarum* is also very heterogeneous and can be divided into several populations (Naumova et al., 2004). Based on a study of 12 isolates, it was shown that the *LAC4* and *LAC12* genes are found specifically in the var. *lactis* strains and confer the ability to ferment lactose (Naumov et al., 2006). Recently, it has been established that these two genes were acquired by introgression from *K. marxianus* (Varela et al., 2019). A population genomic study based on the analysis of the complete genome of 14 *K. marxianus* isolates revealed that dairy and non-dairy strains mainly differ not only by polymorphism within the *LAC12* gene, but also by variation in ploidy level, suggesting that multiple characteristics separate the dairy and non-dairy strains (Ortiz-Merino et al., 2018).

To date, the evolutionary history of the *K. lactis* species remains unclear, and the comparative genomic analysis of the wild and dairy strains was based on a limited number of isolates and only focused on short genomic regions, preventing a complete view of genome evolution and the impact of domestication within this species. Therefore, we sought to study the evolutionary history of this species based on the whole-genome sequence analysis of 42 *K. lactis* strains, originating from both dairy and non-dairy environments. As an interesting alternative model in yeast genetics, *K. lactis* was among the first eukaryotic organisms to have its complete genome sequenced (Dujon et al., 2004). Strain CBS 2359, which belongs to the variety *K. lactis* var. *lactis*, was chosen as reference and the availability of this high-quality 10.6 Mb sequence and its associated annotations allows the establishment of a population genomic analysis. Exploration of the global pattern of polymorphisms allowed to draw a precise view of the phylogenetic relationships between strains, revealing very separated lineages in which individuals are very closely related. Our analyses confirm that the entire dairy lineage is characterized by an introgression of the *LAC4* and *LAC12* genes from *K. marxianus*. However, careful determination and examination of the structure of the introgressed regions revealed that this gene cluster was acquired through multiple and independent introgression events after the divergence of the dairy and wild lineages. In addition, using CNVs, we identified several genes whose presence/absence pattern could indicate a role in adaptation to dairy environments. Overall, our study offers new insights into the evolutionary history of *K. lactis* and the impact of domestication processes on it.

## Results

### *Kluyveromyces lactis* is Characterized by a High Genetic Diversity and a Structured Population

For this study, we gathered a collection of 42 isolates from around the world and coming from diverse ecological niches (supplementary Table S1, Supplementary Material online). Within our collection, 19 strains have been isolated from dairy environments (*e.g*., cheese, buttermilk, or cream) and come mainly from Europe. Natural isolates have been mostly isolated from insects and trees in Asia and North America. We subjected the almost entire collection (41 strains out of the 42, the last one corresponding to the type strain previously sequenced) to short-read whole-genome sequencing, with a mean coverage of 115-fold per sample. The reads associated with each of the 41 samples were mapped to the *K. lactis* reference genome. A total of 1,767,970 reference-based polymorphic positions were detected in the population, 1,594,125 being related to SNPs and 173,845 to small indels.

The SNP dataset was used to evaluate the overall genetic diversity within the species. The average pairwise difference between strains π reaches 2.8 × 10^−2^ (θ_w_ = 3.3 × 10^−2^), which is almost 10-fold higher compared with *S. cerevisiae* (π = 3 × 10^−3^) (Peter et al., 2018). This population-level genetic divergence is, to our knowledge, the highest reported to date within a yeast species. While several species such as *Saccharomyces uvarum* and *Lachancea kluyveri* showed greater diversity compared with *S. cerevisiae* (π = 1.2 × 10^−2^, θ_w_ = 1.2 × 10^−2^ and π = 1.7 × 10^−2^, θ_w_ = 2.1 × 10^−2^, respectively), none were ever mentioned as exceeding 2 × 10^−2^ (Almeida et al., 2014; Friedrich et al., 2015). Interestingly, the diversity within the closely related species, *K. marxianus*, in which dairy and wild strains also coexist, is more than twice lower and has been estimated at π = 1.2 × 10^−2^ (Ortiz-Merino et al., 2018).

**Fig. 1.**
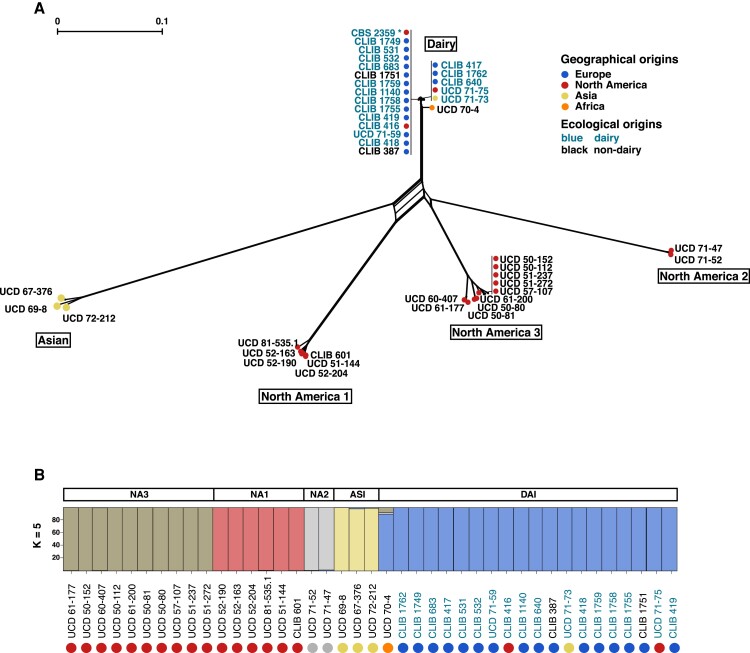
Population structure of 42 *Kluyveromyces lactis* strains. (*A*) Neighbor-net analysis based on 1,594,125 SNPs identified in the surveyed strains. Branch lengths are proportional to the number of sites that discriminate each pair of strains and crossing edges indicate the likely occurrence of recombination. (*B*) Structure of the population, with *K* = 5 populations. Each strain is represented by a vertical bar, which is divided into segments that represent the strain's estimated ancestry proportion in each of the five populations. The circle colors denote the geographical origins of the strains.

This SNP dataset was used for a neighbor-net (SplitsTree) analysis that clusters the strains into five highly separated lineages (fig. 1*A*), within which individuals are closely related (supplementary Table S2, Supplementary Material online). This topology was confirmed by the inference of the phylogenetic relationships between the strains, through the construction of a maximum-likelihood tree, as well as a neighbor-joining tree (supplementary fig. S1, Supplementary Material online). The largest population corresponds to the *K. lactis* var. *lactis* part of the tree and includes 21 strains among which the reference strain and all those associated with dairy products. Excluding UCD 70–4, which was isolated from a winery in South Africa and is significantly more divergent, the average pairwise divergence observed between the strains in this group is 0.06% (supplementary Table S3, Supplementary Material online). The very low genetic divergence in this population is very close to what can be observed, for example, in the sake population of *S. cerevisiae*, with an average divergence of 0.08%, which may be a distinctive sign of domestication (Peter et al., 2018). The *K. lactis* var. *drosophilarum* strains are distributed in four distantly related lineages whose segregation correlates mostly with the geographical origins of the strains (fig. 1*A*, supplementary fig. S2, Supplementary Material online). We named these lineages based on these origins, leading to an Asian cluster and three distinct North-American clusters (NA1, NA2, and NA3). The intra-group mean genetic divergence ranges from 0.26% to 0.78% for the NA2 and NA3 lineages, respectively, which is much higher compared with the dairy lineage. The population structure is in complete accordance with the distribution of the strains on the tree and each group is represented as a clear population for which no admixture has been highlighted (fig. 1, supplementary fig. S3, Supplementary Material online). This suggests the absence of interlineage outcrossing events, which could be attributed to the geographical separation of the isolates. These results confirmed the great heterogeneity within *K. lactis* var. *drosophilarum* and the existence of clear lineages, already suggested by the analysis of small genomic regions (Naumova et al., 2004).

Interestingly, the genetic divergence between the clusters is very high, exceeding 8.5 SNPs every 100 bp between the two most divergent populations (F_ST_ = 0.94), namely the Asian and North America 2 groups (supplementary Tables S4 and S5, Supplementary Material online). Such genetic diversity between the lineages along with the absence of reticulation within the tree (fig. 1*A*) raises the question of reproductive isolation within this species. In a previous study, isolates from different lineages were crossed and the spore viability was estimated (Naumov & Naumova, 2002). While the intra-lineage crosses led to fertile hybrids, the intercluster crosses led to very low viability (< 45%). The recent release of the complete genome of the type strain of *K. lactis* var. *drosophilarum* (CBS 2105 = CLIB 601), which belongs to the NA1 cluster, showed large chromosomal rearrangements compared with the type strain of *K. lactis* var. *lactis* (CBS 2359 = CLIB 210), the reference isolate of the dairy population (Varela et al., 2019). These types of evolutionary events could lead to reproductive isolation, as could the strong genetic divergence associated with the allopatric distribution of taxa that could lead to incompatible alleles. Taken together, these observations cast doubt on the current delineation of *K. lactis* as a species, or at the very least, point to an early stage of a speciation process.

### Ploidy Level and Chromosome Number Variation

Systematic analysis by flow cytometry confirmed that the *K. lactis* species is primarily a haplontic species because all strains have a haploid profile, with the exception of UCD 57–107, for which a diploid profile was obtained (supplementary Table S1, Supplementary Material online). UCD 57–107 showed a very low proportion of heterozygous SNPs (<1%), comparable to what was observed for haploid strains, and therefore, was considered as homozygous. The read coverage profiles along the reference genome revealed that our collection was devoid of aneuploidy. Based on 10-kb sliding windows, a total of 14 segmental duplications were detected in seven isolates (from 1 to 7 segmental duplications per isolate) (supplementary Table S1, Supplementary Material online), all chromosomes being affected, but chromosome 2. Chromosome 4 was the most affected, with four strains carrying a segmental duplication on its right arm. The lack of variability in the level of ploidy in *K. lactis*, as well as the absence of variability in the number of chromosomes contrast with what has recently been observed in the other yeast species. In the case of its sister species, *K. marxianus*, ploidy level has been shown to separate dairy and non-dairy strains, the dairy strains being diploid or triploid, and the non-dairy strains being haploid (Ortiz-Merino et al., 2018). Higher ploidy levels and aneuploidies were also recently described as enriched in some domesticated clades of *S. cerevisiae* (Peter et al., 2018). Altogether, our results suggest that these mechanisms are not driving evolutionary processes related to the dairy environment in *K. lactis*.

### Gene Content, Copy Number Variation and Domestication

**Fig. 2.**
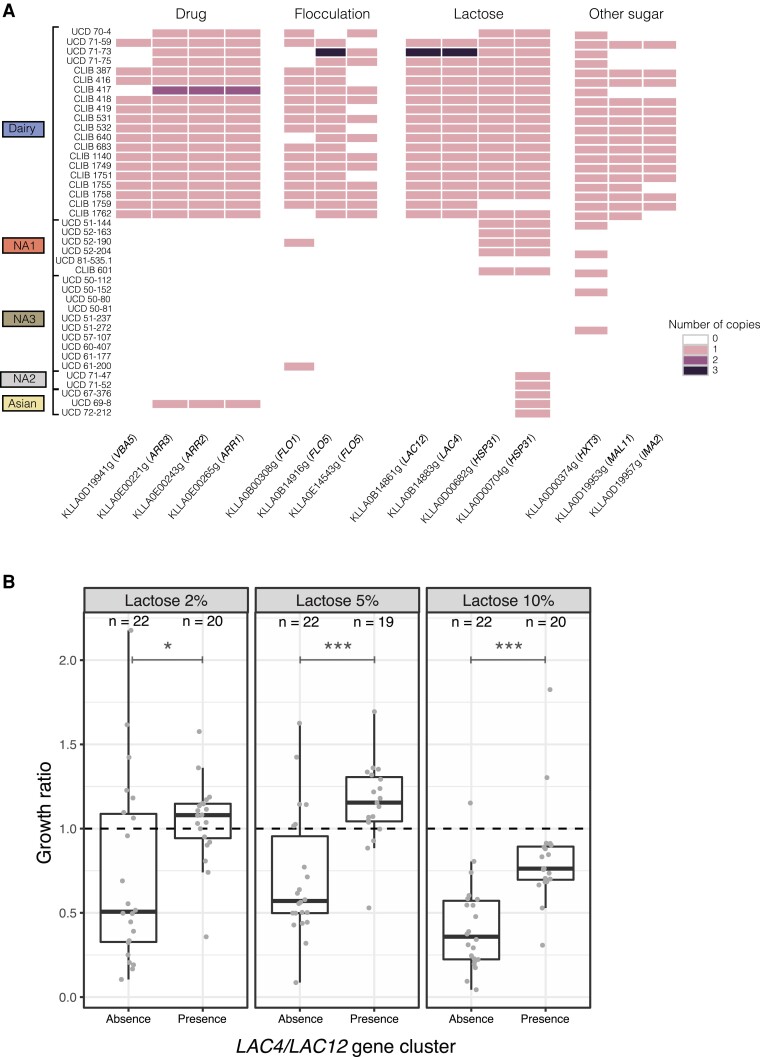
Diversity in terms of gene copy number within the population. (*A*) Number of copies of a subset of genes having differential profiles between the dairy and non-dairy isolates. Strains are organized by population. (*B*) Distribution of the growth ratio, that is the max OD in the tested conditions normalized by the max OD obtained in glucose 2% condition, of the *Kluyveromyces lactis* strains with increasing concentration of lactose, according to the presence or the absence of the *LAC4/LAC12* gene cluster. Wilcoxon–Mann–Whitney test was applied to assess the significance of the growth ratio difference between the two groups of strains. The level of significance is indicated as follows: ns, not significant, *P ≤ 0.05, **P ≤ 0.01, ***P ≤ 0.001.

The *LAC4* and *LAC12* genes, which encode ß-galactosidase and lactose permease, respectively, have been identified for more than a decade as controlling the fermentation of lactose in *K. lactis* var. *lactis,* as these two genes were absent in strains unable to ferment lactose (Naumov et al., 2006). Here, the presence of these genes was investigated across the entire collection, and as expected, they are found to be absent in all strains that did not belong to the dairy cluster (fig. 2*A*). Within the dairy cluster, a single strain, UCD 70–4, was found not to carry these genes. This strain was isolated in winemaking equipment and has been described as closely related to dairy strains, but without the ability to metabolize lactose (Naumov et al., 2006). This characteristic could be attributed either to a loss of these genes or to the absence of introgression events. The outlier position of this isolate compared with the dairy clean lineage within the tree suggests that it might actually predate the introgression event and be a close relative to the dairy ancestor that underwent the introgression event.

To test the ability of our strain collection to assimilate lactose, the growth of the 42 natural strains was recorded in complete medium alone and with increasing concentrations of lactose (2%, 5%, and 10%), using a microcultivation approach. These results clearly demonstrate better growth capacities under lactose conditions for the strains having the *LAC4* and *LAC12* genes, this difference being more significant at 5% and 10% of the lactose concentration (fig. 2*B*). This growth advantage is most likely related to the presence of this gene cluster; however, we cannot exclude that some other genes contribute to the phenotype.

While the main driver of lactose uptake is obviously related to the presence of this gene cluster, the availability of sequencing data for a large *K. lactis* collection paves the way for a more systematic view of the variants that discriminate populations at the genomic level. To this end, gene CNV were first investigated based on read mapping to the *K. lactis* var. *lactis* reference with Control-FREEC (Boeva et al., 2012). Within the whole population, we detected 1,406 gene gains and 2,027 gene losses related to 937 unique genes annotated on the reference genome (supplementary fig. S4*A*, Supplementary Material online). However, the distribution of CNVs is highly affected by segmental duplications, in particular, for the UCD 50–81 and UCD 50–80 strains, for which 270 and 146 specific gene gains are, respectively, reported (supplementary fig. S4*B*, Supplementary Material online). To identify genes with differential profiles between the dairy and non-dairy populations, we applied a two-sample Kolmogorov–Smirnov test and selected genes with a *P* value lower than 0.05. This led to the identification of 87 genes, most of which share a common profile, that is predominantly present in the dairy population and absent in the others (supplementary Table S6, Supplementary Material online). Among this set of genes, *LAC4*, *LAC12,* and *FLO*, which are part of the regions introgressed from *K. marxianus*, are the genes that are found systematically in the dairy strains only. A single strain, UCD 71–73, showed a higher number of copies of these genes, suggesting that their duplication is not a driving factor for dairy environment adaptation. Some interesting genes are also present in this set of 87 genes. For example, two genes, KLLA0D00682g and KLLA0D00704g, are annotated as similar to the *HSP31* gene of *S. cerevisiae*, known to be involved in oxidative stress resistance and which methylglyoxalase activity converts methylglyoxal to D-lactate (Tsai et al., 2015). Certain genes involved in the metabolism of other sugars, such as the maltose transporter *MAL11*, the isomaltase *IMA2,* and the hexose transmembrane transporter *HXT3,* and in flocculation were also detected in most of the dairy strains although they were not found in the majority of wild strains. These genes have already been found in bread and cheese *S. cerevisiae* strains and also play an important role during dairy processes (Legras et al., 2018). Finally, genes involved in drug resistance, including the plasma membrane protein *VBA5* and the *ARR* genes (*ARR1-3*) linked to the arsenate resistance, showed similar profiles. These genes have already been identified as playing a major role in arsenate resistance in *S. cerevisiae* and could, therefore, play a role during industrial processes (Bobrowicz et al., 1997; Peter et al., 2018).

### Multiple Introgression Events of the *LAC* Cluster in the Dairy Population

While the presence of the *LAC4/LAC12* gene cluster has been identified for over a decade as controlling the fermentation of lactose in *K. lactis* var. *lactis* (Naumov et al., 2006), it was only recently shown that this gene cluster was acquired by introgression from the sister species *K. marxianus* (Varela et al., 2019). This event also leads to the integration of a flocculin gene that has since acquired frameshift mutations. To better understand the structure and the evolution of this introgressed region, we sought to assemble the genomes of all strains of the dairy population, based on our Illumina sequencing data. As expected, the three-gene cluster was detected in the Illumina assemblies of all strains isolated from dairy environments. This gene cluster was, however, not detected in strain UCD70-4, isolated from a South African winery, which is more distant from all other isolates of this lineage. Two scenarios can be considered here: either the acquisition of the introgressed region occurred after the divergence between the ancestor of the UCD 70–4 and the other dairy strains which would imply that the divergence between *K.* var. *drosophilarum* and var. *lactis* populations preceded the introgression event, or this strain lost the introgressed region during its evolutionary history.

**Fig. 3.**
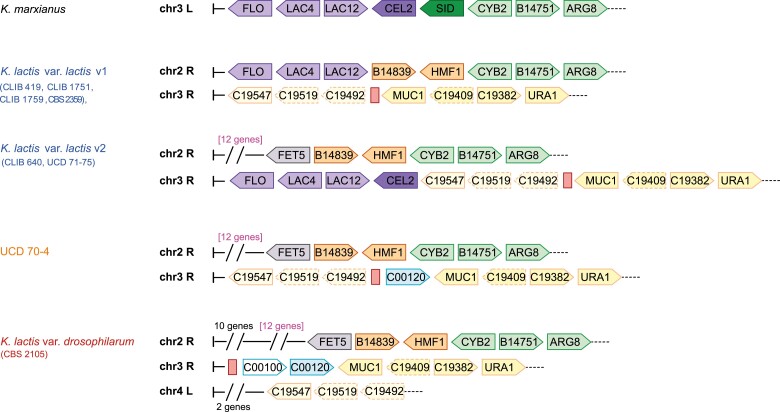
Chromosomal structure of the introgressed *LAC4*/*LAC12* gene cluster in several *Kluyveromyces lactis* and *Kluyveromyces marxianus* backgrounds. On this schematic representation of the chromosomal regions related to the introgression events that occurred from *K. marxianus* into the genome of the *K. lactis* dairy strains, *K. lactis* var. *lactis* v1 referred to the dairy reference strain, *K. lactis* var. *lactis* v2 referred to the newly detected cluster in some dairy strains. The name of the strains is colored according to their origins: blue for European dairy, orange for African non-dairy and red for North-American non-dairy. L and R referred to the left and right extremities of the chromosomes, respectively. Vertical bars (on the left) represent the end of the chromosomes, whereas the black dotted lines (on the right) indicate that the chromosomes continue. Genes in syntenic regions share the same color and dotted boxes are associated with pseudogenes. Red boxes indicate the presence of LTR elements.

Surprisingly, an additional gene coming from *K. marxianus*, *CEL2*, was detected next to the introgressed regions for five isolates, namely CLIB 417, CLIB 640, CLIB 1762, UCD 71–73, and UCD 71–75. The *CEL2* gene is the first neighbor of *LAC12* on the *K. marxianus* genome from which the introgressed region originates (fig. 3). This observation raised the question of the evolutionary history of introgressed regions and whether a larger region encompassing *CEL2* was first introgressed into the common ancestor of dairy strains and then lost in some isolates, or whether multiple introgressions have occurred within the population. The very low sequence divergence of this region in the dairy strains does not allow us to discriminate between these two scenarios based on gene sequence analysis. In this context, we sought to explore the genomic context of these regions. The high level of fragmentation of Illumina assemblies prevented us from having a clear view of the genomic structure within these isolates. This motivated the long-read sequencing of six isolates from the dairy population: two strains harboring the *CEL2* gene (CLIB 640 and UCD 71–75), three strains lacking this gene (CLIB 1751, CLIB 1759, and CLIB 419), as well as the non-dairy strain UCD 70–4 (supplementary Table S1, Supplementary Material online). Chromosome-level assemblies were generated for all of them and made it possible to highlight two different genomic structures for this introgressed region (fig. 3). Indeed, the three-genes cluster shared all the same chromosomal location as in the reference strain, that is at the end of the right-arm of chromosome 2 (version v1), whereas the four genes clusters were detected at the end of the right-arm of chromosome 3 for both strains (version v2) (fig. 3). Comparative analysis of these chromosomal structures suggested that several independent introgression events could have occurred from *K. marxianus* into the *K. lactis* dairy genomes. Indeed, the ends of the right arm of chromosome 2 are entirely syntenic between UCD 70–4 and the *K. lactis* var. *lactis* v2 background. This region is also mostly syntenic with that of *K. lactis* var. *drosophilarum* (Varela et al., 2019), and the difference is based on the presence of ten additional genes in the very subtelomeric regions that are known to be highly variable in yeast genomes. It should be noted that the synteny between the *FET5* and *KLLA0B14839* genes is conserved in these three cases. Consequently, in case of a single introgression event of the *LAC* cluster within the dairy group, the one observed in the *K. lactis* var. *lactis* v2 background should have been the ancestral event and would have preceded the accumulation of chromosomal rearrangements that would have led to the *K. lactis* var. *lactis* v1 chromosomal structure. Nevertheless, the complete synteny observed between UCD 70–4 and *K. lactis* var. *lactis* v1 background at the end of the right arm of chromosome 3, from the telomeric region to the LTR repeats, tends not to support this scenario. This suggests that at least two independent introgression events occurred within the *K. lactis* var. *lactis* population.

Finally, the synteny observed at the right-end of chromosome 2 and that of chromosome 3 between UCD 70–4 and *K. lactis* var. *lactis* v2 supports the fact that the divergence of the so-called dairy and the other clades predates the acquisition of dairy properties. In addition, the branching of UCD 70–4 compared with all strains in which the introgressed region was detected demonstrates that this isolate can be considered as the closest non-dairy, relative to the ancestral strains into which the introgressions occurred.

### Transposable Element Content is Clade Specific

It has recently been shown that the evolution of the transposable element (TE) content follows the population structure in *S. cerevisiae* and is deeply affected by clade-specific events such as introgressions (Bleykasten-Grosshans et al., 2021). The genome-wide content of TEs was investigated through short-read mapping on finely selected TE queries originating from both *K. lactis* and *K. marxianus*. The TE landscape clearly shows that the *K. lactis* populations display specific TE repertoires, which are mainly delineated by population-specific TE (supplementary fig. S5, Supplementary Material online). When a TE is present in a given isolaIe, it is found in most of the isolates of the related population and absent in the other populations. For example, a TE family similar to Tkl-3 is specific to the Asian groups, whereas TE families similar to Tkl-1, Rkl-3, or Rkl-4 are specific to dairy isolates. This exclusive distribution of population-specific TEs that are unrelated contrasts with the population-specific landscape that has been observed in *S. cerevisiae*, which is characterized by both population-specific TEs from the same TE family (i.e., population-specific Ty1 sequence variants) and combinations of TEs shared by multiple populations. Interestingly, none of the *K. marxianus* TE were detected in our *K. lactis* isolates. The dairy population carried the lowest TE content. In most cases, only one copy per genome is present, and in many strains, only partial Tkl-1 and Tkl-2 were detected, suggesting that either the corresponding TE is truncated or that they present segments of sequence too divergent to allow the mapping of reads. There is a striking exception represented by the CLIB418 isolate, which contains approximately a hundred of Tkl-1 copies. These copies are clearly the result of a transposition burst, as supported by the uniform shape of the coverage profile indicating that they are very similar.

### Mitochondrial Genome Diversity in *K. lactis*

The mitochondrial genome of *K. lactis* is 40.3 kb long and contains the same set of eight protein-coding genes as *S. cerevisiae*. These genes encode three subunits of the ATP synthase complex (*ATP6*, *ATP8*, *ATP9*), the apocytochrome b (*CYTB*), three subunits of the cytochrome c oxidase complex (*COX1*, *COX2*, *COX3*), and a ribosomal protein (*VAR1*). We sought to explore mitochondrial genome diversity within our collection through genome assemblies, but their high level of fragmentation prevents comparison at the whole mitochondrial genome level. Complete gene coding sequences could only be recovered for four genes (*ATP6*, *COX2*, *COX3,* and *CYTB*) across the 41 isolates. Interestingly, the *ATP6* and *COX2* genes were recently identified as efficiently recapitulating the population structure and phylogeny inferred from mitochondrial gene sequences in *S. cerevisiae* (De Chiara et al., 2020). For this reason, we confidently thought that limiting our analysis to these four genes would still help to better understand the mitochondrial genome evolution.

The genetic diversity related to these four genes was lower compared with the nuclear coding genes, for which a mean π value of 0.023 was observed. However, mitochondrial genetic diversity varies a lot among genes (supplementary Table S7, Supplementary Material online): *COX2* and *ATP6* showed very low genetic diversities, with π values of 0.0011 and 0.0036, respectively, whereas *COX3* and *CYTB* had much higher genetic diversities, with π values of 0.0160 and 0.0165. These results are in contrast with what was observed in *S. cerevisiae*, for which *COX2* and *ATP6* were among the more highly divergent genes (with π values of 0.0166 and 0.0108, respectively) and *COX3* and *CYTB* were among the less divergent genes (with π values of 0.0072 and 0.0048, respectively). These four gene sequences were concatenated and used to construct a neighbor-joining tree (supplementary fig. S6, Supplementary Material online). Interestingly, the phylogeny is broadly consistent with that obtained from nuclear sequences, with the exception of isolates from the two North-American groups, which are completely mixed, revealing some mito-nuclear discordance (supplementary fig. S6, Supplementary Material online). This contrasts with what was recently observed for *S. cerevisiae* (De Chiara et al., 2020), for which a very discordant evolution of the mitochondrial and nuclear genomes was observed, due to outbreeding and recombination events between the parental mitochondrial genomes. These results suggest the absence of natural crosses between isolates from different populations, with the exception of North-American strains which may not have been geographically isolated.

## Conclusion

Overall, our study represents the first population genomic survey focusing on a large population of *K. lactis* isolates. This species is of interest as it is partially domesticated, which led to differential genome evolution across the various identified populations. By sequencing the whole genome of 41 *K. lactis* isolates (*K. lactis* var *lactis* and var *drosophilarum*), we found the highest intraspecific genetic divergence ever reported for a yeast species. Compared with other yeast species explored to date, *K. lactis* presents a nucleotide diversity (estimated at 2.8 × 10^−2^), which is on an average, 2-fold higher (e.g., for *S. cerevisiae*, *S. uvarum*, *L. kluyveri*, π is approximately ∼ 4 × 10^−3^, ∼1.2 × 10^−2^, and ∼1.7 × 10^−3^, respectively) (Schacherer et al., 2009; Almeida et al., 2014; Friedrich et al., 2015). Based on the SNP diversity, the isolates grouped into five distinct clusters with the dairy group (*K. lactis* var *lactis*) being well separated from the wild ones (*K. lactis* var *drosophilarum*). However, the four wild populations are also very distant as the SNP-based divergence between these clusters is above 3% and can exceed 8% between the most divergent ones, namely the Asian and North America 2 groups. These observations, to which can be added the detection of large chromosomal rearrangements between several strains, raise the question of the delineation of *K. lactis* as a species and probably suggest an early stage of a speciation process. This hypothesis is also supported by the presence of reproductive isolation between all these populations, as previously described (Naumov & Naumova, 2002). While the intrapopulation crosses lead to fertile hybrids, the intercluster crosses lead to very low viability (<45%).

Our study also provided a better understanding of the domestication process of the dairy population of *K. lactis* (*K. lactis* var *lactis*). Recently, there has been a particular interest in exploring the domestication processes of different species found in milk and dairy products such as *K. marxianus* (Ortiz-Merino et al., 2018), as well as those involved in the maturation of cheese such as *P. roqueforti* and *P. camemberti* (Cheeseman et al., 2014; Ropars et al., 2020). Even if certain characteristics are common to these different processes of domestication, they all have their specificity. Here, we found that the nucleotide variability within the *K. lactis* dairy population is limited. Indeed, the maximum pairwise diversity ranges from 0.23% to 0.79% for the wild clusters, whereas it does not exceed 0.06% between the strains presenting dairy capacities. This reduced genetic diversity within the dairy cluster seems to be a clear feature of the domestication process in *K. lactis*.

As already shown for species isolated from dairy products or used in cheese maturation, horizontal gene transfers or introgressions are key players of domestication events (Cheeseman et al., 2014; Ropars et al., 2020; Varela et al., 2019). Although the impact of the introgression of the *LAC4*/*LAC12* genes cluster from *K. marxianus* was already assessed, our study also highlighted some interesting evolutionary features regarding this aspect in the *K. lactis* dairy population. We first showed that the divergence between the *K. lactis* var*. lactis* and *K. lactis* var*. drosophilarum* isolates occurred before the acquisition of the dairy abilities, that is the introgression of the *LAC4*/*LAC12* genes cluster. Our results also suggest that strain UCD70–4, which is part of the dairy cluster but without dairy capabilities, is the closest sequenced non-dairy isolate to the ancestral strains in which introgressions occurred. Based on very limited sequencing regions (5.8S rRNA, ITS1, ITS2), a European wild population was described as closely related to UCD 70–4 (Naumova et al, 2004). Unfortunately, none of this strain was part of our collection. A more detailed analysis of their whole-genome sequences could help in clarifying the small-scale evolution at this peculiar lineage level. Additionally, the genomic resolution we obtained using long-read sequencing and de novo assemblies allowed us to suggest that several independent introgression events occurred within this species.

Finally, it should also be noted that in *K. lactis*, no real variability in terms of ploidy was observed. Aneuploidies and CNVs are not predominant. This observation contrasts with other yeast species that have undergone domestication processes, in particular with *K. marxianus*, for which ploidy variation separates the dairy and non-dairy strains (Ortiz-Merino et al., 2018), but also *S. cerevisiae* for which some domesticated populations, such as the beer isolates, have been described as having a higher ploidy level (Peter et al., 2018; Saada et al., 2022).

Overall, our study sheds new light on the domestication of the dairy population of *K. lactis*. In addition, it clearly shows once again that even if some characteristics are shared, each domestication process is specific and unique to each domesticated population.

## Materials and Methods

### Studied Strains

A collection of 42 *K. lactis* strains was compiled for this study (supplementary Table S1, Supplementary Material online) with the aim to maximize the ecological and geographical origins of the strains. The samples were mostly purchased from the Phaff and the CIRM collections. Our final dataset contains 17 strains directly related to dairy processes, which originate mostly from Europe. While the number of dairy strains is high, some share closely related origins, which could impact the diversity among these isolates. The wild strains were mostly isolated from insect and tree exudates in North America and Asia.

### Illumina Sequencing and Polymorphisms Detection

To obtain genomic DNA for 41 strains sequenced in the context of this study, isolates were grown overnight at 30 °C in 20 mL of YPD medium to early stationary phase before cells were harvested by centrifugation. Total genomic DNA was subsequently extracted using the MasterPure Yeast DNA purification kit (Cat No MPY80200) according to the manufacturer's instructions. For all genomes, 280-bp insert libraries were produced and sequenced on an Illumina HiSeq 2000 platform. A 100-bp paired-end reads were generated.

For each strain, reads were mapped to the reference genome of the *K. lactis* var. *lactis* type strain CBS 2359 (GenBank accession numbers CR382121 to CR382126) using BWA mem v.0.7.15 (Li & Durbin, 2009) with default parameters. Alignments were then successively cleaned using the samtools fixmate function v.1.3.1 (Li et al., 2009), GATK v.4.0.11 realignment function (McKenna et al., 2010), and Picard MarkDuplicates function v.1.140 (broadinstitute.github.io/picard). The resulting BAM files were then used to detect SNPs and small indels using GATK HaplotypeCaller with a requested ploidy set to one. GVCF files obtained were finally concatenated into one single GVCF using GATK CombineGVCFs and GenotypeGVCFs functions, allowing to produce correct genotype likelihoods and regenotype the newly merged record.

### Oxford Nanopore Sequencing

Yeast cell cultures were grown overnight at 30 °C in 20 mL of YPD medium to early stationary phase before cells were harvested by centrifugation. Total genomic DNA was then extracted using the QIAGEN Genomic-tip 100/G according to the manufacturer's instructions. The extracted DNA was barcoded using the EXP-NBD104 native barcoding kit (Oxford Nanopore) and the concentration of the barcoded DNA was measured with a Qubit® 1.0 fluorometer (Thermo Fisher). The barcoded DNA samples were pooled with an equal concentration for each strain. Using the SQK-LSK109 ligation sequencing kit (Oxford Nanopore), the adapters were ligated on the barcoded DNA. Finally, the sequencing mix was added to the R9.3 flowcell for a 48 h run. Base calling was performed with Guppy (v2.3.5) and raw fastq files were treated with porechop (v0.2.3) to remove both adapters and barcodes.

### Tree Building, Divergence Calculation and Structure Analysis

We inferred phylogenetic relationships among the 41 isolates using the dataset of 1,594,125 SNPs in a maximum-likelihood framework with IQ-Ttree2 (Minh et al., 2020), with TVM + F as a model. This latter was chosen as the best-fit model by BIC through ModelFinder (Kalyaanamoorthy et al., 2017). These polymorphic positions were also used for the neighbor-net analysis which was performed with SplitsTree5 software via the SplitsNetworkViewer.

Isolate relationships were also assessed through the construction of a distance tree using the BioNJ algorithm (Gascuel, 1997) provided in the SplitsTree4 software (Huson & Bryant, 2006). To that end, a sequence representative of each strain was constructed by inferring SNPs within the reference chromosomes, that were then merged into a single 10.7 Mb sequence. All strain sequences were given as input to SplitsTree.

These sequences were also leveraged to estimate the pairwise divergence between each strain as the ratio between the number of nonequal positions between the two considered strains and the total size of the genome.

Finally, these sequences were used for the inference estimation of the number of population clusters through STRUCTURE software, version 2.3.4 (Pritchard et al., 2000). We ran the software independently with a number of populations, K ranging from 2 to 6, using the admixture model with a burn-in period of 100,000 and 500,000 replicates.

### Calculation of Population Genetic Statistics

In order to get an estimate of the nucleotide diversity at the population level, π, the average pairwise nucleotide diversity θw, the proportion of segregating sites and Tajima's D value, which represents the difference between π and θw was computed. Multiple alignments of the concatenated chromosomes that were representative of the isolates were submitted to variscan (Vilella et al., 2005) with options runmode set to 12 and usemuts set to 1.

### Determination of Copy Number Variants

The detection of CNVs related to the *K. lactis var. lactis* reference and affecting each strain was performed by running Control-FREEC (Boeva et al., 2012), version 10.6 on their respective BAM files. The program was used with the following parameters: breakPointThreshold = 0.6, window = 1000, telocentromeric = 600, step = 200, ploidy = 1, minExpectedGC = 0.3, and maxExpectedGC = 0.4. To obtain a count of the number of copies for each genomic feature, Control-FREEC output files were crossed with the reference genome annotations. Features for which at least half of the length was contained in a region whose number of copies deviated from one were considered under CNV. If regions with different coverages overlapped a single feature, the one covering the larger part of the feature was considered.

### Analysis of the Copy Number Variants

To identify genes with variable CNV patterns between the dairy and non-dairy varieties, a two-sample Kolmogorov–Smirnov statistic test was applied to each of the 5,076 protein-coding genes annotated in the reference genome. Genes for which distributions differ between both varieties (*P* value < 0.05), were further selected for manual inspection.

### Transposable Element Detection

The strategy used in Bleykasten-Grosshans et al. (2021) was adapted for this study. Briefly, a set of 23 sequences originating from *K. lactis* and *K. marxianus* was defined as the representative dataset. Among them, nine sequences belong to the Class II Rover elements from the hAT superfamily described in Sarilar et al. (2015). The other 14 query sequences are similar to Class I LTR elements from the Copia superfamily. The Class I query sequences were named Tkl or Tkm (for Ty *K. lactis* and Ty *K. marxianus*, respectively), according to the nomenclature used in Neuvéglise et al., (2002). Similarly, the Class II sequences were named Rkl or Rkm (for Rover *K. lactis* and Rover *K. marxianus*, respectively). The Illumina reads of the 41 strains were mapped against this dataset and the coverage profiles were manually inspected. Only eight *K. lactis* queries showed significant coverage that allowed estimating the number of copies of the considered element in different strains.

### Whole-Genome Assembly Construction

Illumina paired-end reads assemblies were constructed with Abyss (v.2.0.2) (Simpson et al., 2009) with the option “-k 64“.

For the long-read assemblies, the Oxford Nanopore fastq files were downsampled with Filtlong (v0.2) (https://github.com/rrwick/Filtlong) to get a 40X coverage per strain (options –min_length 1000 –mean_q_weight 10). The downsampled datasets were then independently assembled with SMARTdenovo (Liu et al., 2021), with the options “-c 1 -k 16 -J 5000 -e zmo”.

### Strain Phenotyping and Growth Quantification

The 42 *K. lactis* strains were inoculated into flat bottom 96-well microplates (Nunclon, Thermo Fisher) containing 150 μL of YPD (Yeast extract 1% Peptone 2% Dextrose 2%) and incubated overnight at 30 °C. Precultures were washed five times in sterile MilliQ water to eliminate residual glucose. After the last washing step, cells were resuspended in sterile MilliQ water and transferred with a MicroPlate Pin Replicator in a 96-well microplate containing 150 μL of synthetic complete media (yeast nitrogen base with ammonium sulfate 6.7 g/L, SC amino acid mixture 2 g/L) with either glucose (2%) or lactose (2%, 5%, or 10%) as the carbon source. Optical density (OD) at 600 nm was measured every 10 min during 48 h at 30 °C using the microplate reader TECAN Infinite® 200Pro. Before each measure, microplates were shaken for 400 s with orbital shaking (87.6 rpm) and 200 s with linear shaking (135.6 rpm) to ensure proper yeast suspension and accurate measure.

The increase in OD as a function of time was modeled by local polynomial regression fitting with the R-loess function setting the span parameter to 0.45. The growth ratio corresponds to the top OD rate in 1h windows in the conditions of interest normalized by those obtained on glucose media.

## Supplementary Material

evad004_Supplementary_Data

## Data Availability

All sequencing data generated in this study have been deposited in the European Nucleotide Archive under the accession number PRJEB29566 for Illumina reads and PRJEB48853 for MinION reads.
